# Cannabidiol Reduces Intestinal Inflammation through the Control of Neuroimmune Axis

**DOI:** 10.1371/journal.pone.0028159

**Published:** 2011-12-06

**Authors:** Daniele De Filippis, Giuseppe Esposito, Carla Cirillo, Mariateresa Cipriano, Benedicte Y. De Winter, Caterina Scuderi, Giovanni Sarnelli, Rosario Cuomo, Luca Steardo, Joris G. De Man, Teresa Iuvone

**Affiliations:** 1 Department of Experimental Pharmacology, University of Naples FEDERICO II, Naples, Italy; 2 Department of Human Physiology and Pharmacology V. Erspamer, University of Rome “La Sapienza”, Rome, Italy; 3 Department of Clinical and Experimental Medicine, University of Naples FEDERICO II, Naples, Italy; 4 Laboratory of Experimental Medicine and Pediatrics, Division of Gastroenterology, University of Antwerp, Antwerp, Belgium; 5 Endocannabinoid Research Group, Pozzuoli, Italy; Sapienza University of Rome, Italy

## Abstract

Enteric glial cells (EGC) actively mediate acute and chronic inflammation in the gut; EGC proliferate and release neurotrophins, growth factors, and pro-inflammatory cytokines which, in turn, may amplify the immune response, representing a very important link between the nervous and immune systems in the intestine. Cannabidiol (CBD) is an interesting compound because of its ability to control reactive gliosis in the CNS, without any unwanted psychotropic effects. Therefore the rationale of our study was to investigate the effect of CBD on intestinal biopsies from patients with ulcerative colitis (UC) and from intestinal segments of mice with LPS-induced intestinal inflammation. CBD markedly counteracted reactive enteric gliosis in LPS-mice trough the massive reduction of astroglial signalling neurotrophin S100B. Histological, biochemical and immunohistochemical data demonstrated that S100B decrease was associated with a considerable decrease in mast cell and macrophages in the intestine of LPS-treated mice after CBD treatment. Moreover the treatment of LPS-mice with CBD reduced TNF-α expression and the presence of cleaved caspase-3. Similar results were obtained in *ex vivo* cultured human derived colonic biopsies. In biopsies of UC patients, both during active inflammation and in remission stimulated with LPS+INF-γ, an increased glial cell activation and intestinal damage were evidenced. CBD reduced the expression of S100B and iNOS proteins in the human biopsies confirming its well documented effect in septic mice. The activity of CBD is, at least partly, mediated via the selective PPAR-gamma receptor pathway. CBD targets enteric reactive gliosis, counteracts the inflammatory environment induced by LPS in mice and in human colonic cultures derived from UC patients. These actions lead to a reduction of intestinal damage mediated by PPARgamma receptor pathway. Our results therefore indicate that CBD indeed unravels a new therapeutic strategy to treat inflammatory bowel diseases.

## Introduction

Despite the ancient assumption that enteric glial cells (EGC) may serve as a mere mechanical support for enteric neurons, nowadays the knowledge on these cells is consistently expanded. EGC play a fundamental role in the maintenance of gut homeostasis since they assure the correct trophism of vicinal neurons in the myenteric plexus [1] and actively participate in the course of intestinal inflammation [2] where they appear as first defensive line against pathogens [3].

Enteroglial cells share analogue features with glial cells in the brain. EGC play important functions in the maintenance of the enteric nervous system (ENS) homeostasis, but they may also proliferate and be activated in response to injury and inflammation undergoing reactive gliosis (entero-gliosis), a dynamic process [4]. Enteric astroglial and microglial cells release neurotrophins, growth factors and cytokines cross-talking with other infiltrating immune cells such as macrophages, neutrophils and mast cells [5], [6], [7].

Abnormalities in the enteroglial network were described in the intestinal mucosa of patients with inflammatory bowel diseases (IBD) [8], measures as the reactive enteric gliosis, i.e. the massive over-expression and secretion of S100B protein, a cell-specific astroglial derived signalling molecule [9]. The activation of EGC is therefore regarded as a general alteration of the whole enteric nervous system homeostasis. S100B protein, which is released by enteric glial cells, emerges as a pivotal signal molecule that extensively participates in the onset and in the progression of the inflammatory status as it orchestrates a wide range of signal activation pathways, directly correlated with the severity of gut degenerative processes [8].

Molecules which may counteract intestinal inflammation targeting EGC could represent putative novel approaches to amplify the current pharmacological tools to treat gut inflammatory diseases. In this sense, a huge amount of data produced in the recent years demonstrated that cannabidiol (CBD) the non-psychotropic cannabinoid deriving from *Cannabis Sativa*, appears as a very promising compound because of its antinflammatory, antioxidant and anti-apoptotic effects in different models of CNS inflammation [10]. It was shown that CBD exerts its pharmacological activity targeting reactive astroglia and this results in a very efficient reduction of the neuroinflammatory/neurodegenerative status both *in vitro* and *in vivo* models of neuropathologies [11].

To date, the effect of CBD on enteric gliosis which occurs during acute and chronic gut inflammation has not yet been evaluated. Therefore, the present study aims to evaluate: (a) the effect of CBD on enteroglial-derived S100B protein expression in a mouse model of acute intestinal inflammation and in rectal biopsies derived from patients with ulcerative colitis; (b) the efficacy of CBD to prevent S100B-mediated amplification of inflammatory/immune response through the involvement of other immune cells such as macrophages and mast cells; (c) the anti-apoptotic effect of CBD in course of inflammation.

Moreover, here we aim to identify a specific receptor responsible for CBD action. Therefore, in the present paper we investigated the involvement of PPAR-γ receptor, since recent data suggest that PPAR-γ activation may underlie some of the pharmacological effects of CBD. In particular, it was showed that CBD, causes a time-dependent progressive vasorelaxant effect similar to that of rosiglitazone, a PPAR-γ agonist, and that the effects of CBD were reversed by the presence of the PPAR-γ antagonist, GW9662 [12], [13].

## Methods

### Cultured human intestinal biopsies

The experimental group comprised 18 subjects (median age 49 years, range 33–65) who underwent colonoscopy for colon cancer screening and had normal rectal mucosa morphology. Eight subjects were in good general health without any previous medical or surgical history; 10 subjects were diagnosed ulcerative colitis (UC). In each subject 3 rectal mucosal samples were obtained 1) at onset, at first colonoscopy, 2) before any treatment (acute group) and 3) at the moment of endoscopically diagnosed remission, (quiescent group). All the subjects underwent endoscopic biopsy from the rectum for histological assessment. We utilized an informed consent that all subjects signed. In this consent we explained to the subjects that extra biopsies could be made to perform some research as in this case. Moreover, this study has been approved to ethical committee of University Federico II – Naples. Whole mount biopsy specimens were cultured, according to previous report [5], in Dulbecco Modified Eagle's Medium (DMEM) supplemented with 5% Foetal Bovine Serum (FBS), 2 mM glutamine, 100 U/mL penicillin, 100 µg/mL streptomycin at 37°C in 5% CO_2_/95% air. Biopsies were placed in 24-well plates and, depending upon the experiments, the medium was replaced with fresh medium and tissue was stimulated for 24 h with exogenous LPS (10 µg/ml)+INF-γ (300 U/mL) alone or in the presence or absence of CBD (10^−8^–10^−6^ M), given to the tissue 30 minutes before LPS+INF-γ stimulus. Rectal biopsies from patients with UC in acute phase were cultured in the presence of CBD (10^−8^–10^−6^ M) for 24 h. In same experiments GW9662 10^−7^ M was given together with CBD 10^−6^ M in UC biopsies in acute phase.

### LPS-induced intestinal inflammation

All procedures received approval from the Medical Ethical Committee of the University of Antwerp, Belgium (number of the study is B00-023).

Male Swiss OF1 mice (30–40 g) were fasted from 09.00 AM by removing food pellets but with free access to tap water. At 4.00 PM the mice were weighed and received the first i.p. injection of cannabidiol (10 mg/kg), or the cannabidiol vehicle (10% ethanol, 10% Tween 80, 80% saline), Thirty min later, the mice were divided in a control and LPS group receiving, respectively, an i.p. injection of vehicle or LPS (E. coli 055:B5; 20 mg/kg). Six hours after injection of vehicle or LPS, mice received a second i.p. injection of the drug under study (cannabidiol 10 mg/kg or the vehicle). All drugs were injected in a volume ratio of 10 µl/g bodyweight. Eighteen hours after LPS or vehicle injection, mice were anaesthetized with diethyl ether and killed by exsanguinations [14], [15].

### Preparation of total extracts

For homogenisation, intestinal tissue was placed in ice-cold lyses buffer (20 mM HEPES, 100 mM MgCl_2_, 0.4 M NaCl, 0.5 mM phenylmethylsulphonylfluoride, 15 µg/ml soybean trypsin inhibitor, 3 µg/ml pepstatin A, 2 µg/ml leupeptin, 40 µM benzamidine, 1 mM dithiothreitol, 1% Nonidet P40, 20% glycerol) in a ratio of 0.4 ml per 100 µg of tissue and homogenized at the highest setting for 2–5 min in Polytron PT300 tissue homogeniser. Protein concentration was determined using the BioRad protein assay kit.

### Western blot Analysis

Immunoblotting analysis of protein was performed on cytosolic fraction. Cytosolic extract fraction proteins were mixed with gel loading buffer (50 mM Tris, 10% SDS, 10% glycerol 2-mercaptoethanol, 2 mg bromophenol/ml) in a ratio of 1∶1, boiled for 5 min and centrifuged at 10.000 g for 10 min. Protein concentration was determined and equivalent amounts (50 µg) of each sample were separated under reducing conditions in 12% SDS-polyacrylamide minigel. The proteins were transferred onto nitrocellulose membrane according to the manufacturer's instructions (Bio-Rad Laboratories, Hercules, CA, USA). Depending upon the experiments, the membranes were blocked by incubation at 4°C overnight in high salt buffer (50 mM Trizma base, 500 mM NaCl, 0.05% Tween-20) containing 5% bovine serum albumin; they were then incubated for 1 h with specific anti-MMP9 (1∶100 v/v; Neomarkers, Fremont, CA), anti-TNF-α (1∶500 v/v, Sigma St Louis, MO, USA), anti-S100B (1∶250 v/v, ABCAM, UK); anti-Chymase (1∶500 v/v; Neomarkers, Fremont, CA), anti-iNOS (1∶1000 v/v; BD, Franklin Lakes, NJ USA) and anti-tubulin (1∶1000 v/v, Santacruz, Santa Cruz, CA) primary antibodies followed by incubation with specific horseradish peroxidase (HRP)-conjugate secondary antibody (Dako, Golstrup, DK). The immune complexes were developed using enhanced chemiluminescence detection reagents (Amersham, Italy) according to the manufacturer's instructions and exposed to Kodak X-Omat film. The protein bands on X-ray film were scanned and densitometrically analysed with a GS-700 imaging densitometer (Bio-Rad Laboratories, CA, USA).

### Immunohistochemistry

For MAC3 and cleaved-caspase 3 immunohistochemistry analysis, intestinal tissue deriving from mice were fixed in buffered formalin, embedded in paraffin, and cut into 4 µm-thick serial sections. Sections were stained with the primary MAC3 (1∶200 v/v) or cleaved-caspase 3 (1∶100 v/v) antibodies. After three 5-min washes, the secondary antibody was added and the samples were incubated at room temperature for 20 min. The streptavidin-HRP detection system was added and samples were incubated at room temperature. After three 5-min washes, 50 µl of chromogen was added and the reaction stopped after 1 min in water.

### Histological analysis

Intestinal tissues were fixed in 10% formalin. Thin (0.5 µm) paraffined section were prepared and stained with toluidine blue as previously described [16] and then processed for light microscopy examination. MC counting was performed on five randomly selected sections using an ×100 objective lens.

### Nitrite assay

Nitrite production, the stable metabolites of NO, was measured in 24 hours-cultured biopsies supernatant. The supernatant (0.1 mL) was added to an equal amount of Griess reagent (1% sulphanilamide, 0.1% naphtylendiammine, 2.5% H_3_PO_4_) and allowed at room temperature for 10 min. The absorbance of constituted chromophore was determined using a UV/visible spectrophotometer at 550 nm. Nitrite levels were determined using a sodium nitrite standard curve and expressed as nmol/µg.

### Drugs used

NaCl 0.9% (Plurule®, Baxter, Lessines, Belgium), LPS (*E. coli* serotype 055:B5, Sigma-Aldrich, St Louis, MO, USA), cannabidiol (Tocris Bioscience, Bristol, UK). Cannabidiol was dissolved in 10% ethanol, 10% Tween 80, 80% saline.

### Presentation of results and statistical analysis

For the functional *in vivo* experiments on gastric emptying and the geometric center, values are shown as mean ± SEM for *n* indicating the number of mice used. For statistical analysis, we used two-way ANOVA. The first factor concerned the presence or absence of LPS, the second parameter the drug under study. For post hoc testing we used a one-way ANOVA followed by a Bonferroni post hoc test or a non-paired Student's t-test as appropriate. P values<0.05 were considered to be significant. All data were analysed with the SPSS for Windows software (SPSS Inc., Chicago, IL, USA). For Western blot analysis results were expressed as the mean ± SEM of n animals where each value is the average of responses in duplicate sites. Statistical comparisons were made by one way-ANOVA followed by Bonferroni's test for multiple comparisons. P<0.05 was considered to be significant. Data were analyzed with GraphPad Instat.

## Results

### Effect of CBD in LPS- induced enteric glial cell activation in the mouse intestine

**Figure 1:**
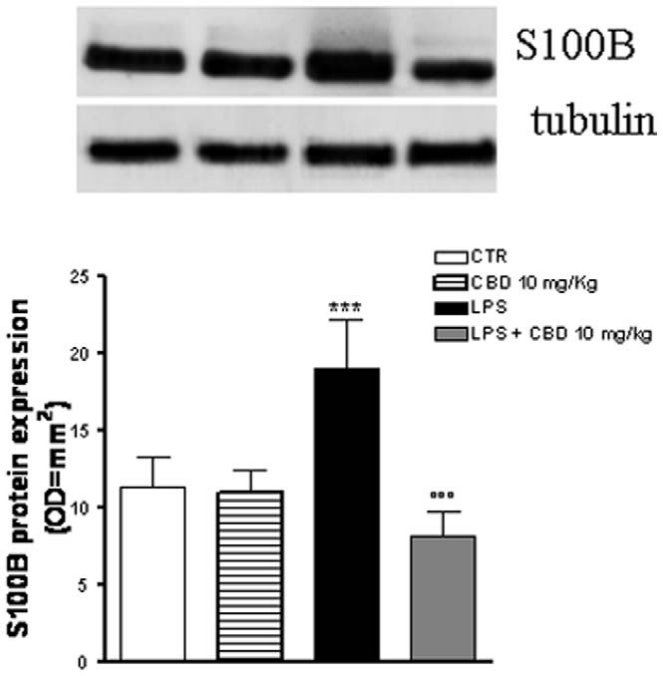
Effect of CBD on glial cell hyperactivation in septic mice. The intraperitoneal administration of CBD in septic mice significantly reduced S100B protein expression. Results are expressed as mean ± SEM of 3 experiments. ***p<0.001 vs. saline; °°°p<0.001 vs. LPS alone.

Immunoblotting analysis of the protein S100B, considered a marker of glial cells since it is exclusively localized in glial cells, revealed that S100B expression was significantly increased in intestinal tissues from LPS-treated mice compared to control mice (Fig. 1). Treatment of control mice with CBD had no effect on S100B expression. However, when mice receiving LPS were treated with CBD, S100B expression was significantly reduced to control values (Fig. 1).

### Effect of CBD in LPS-induced mast cell activation in the mouse intestine

**Figure 2:**
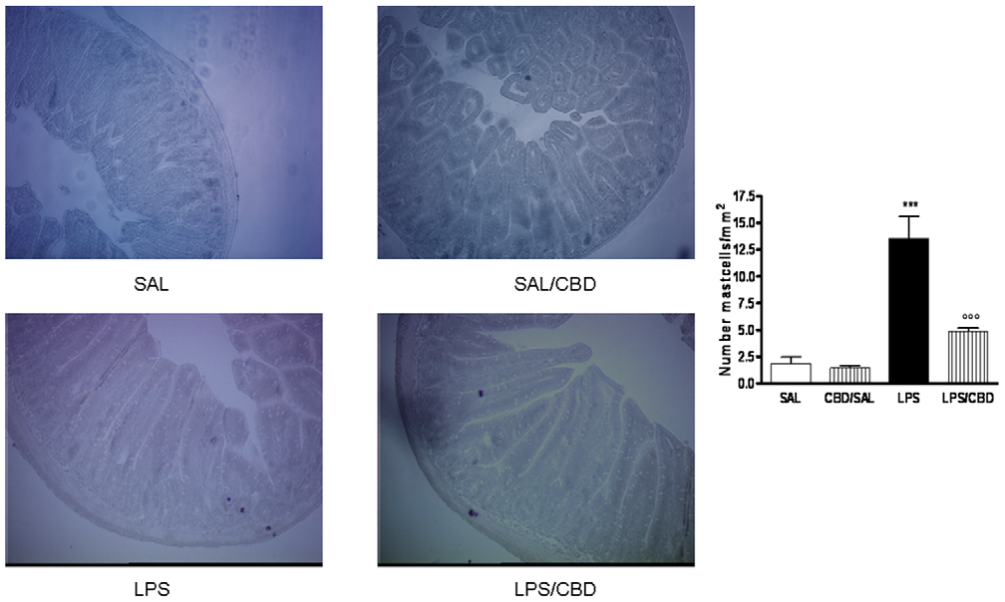
Effect of CBD on mast cell activation in septic mice. (A) MC presence in intestinal tissue was evaluated in representative histological analysis of paraffined tissue stained with Toluidine blue. Fields are representative of 3 separate experiments. Original magnification, 100×.

**Figure 3:**
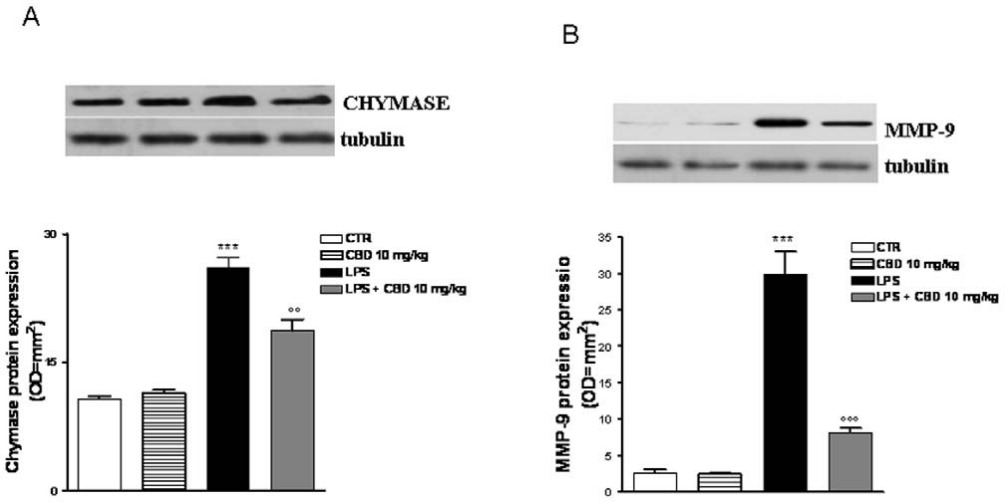
Effect of CBD on mast cell derived. (A) chymase protein expression, (B) MMP-9 protein expression. Results are expressed as mean ± SEM of 3 experiments ***p<0.001 vs. saline; °°°p<0.001 vs. LPS alone Representative Western blot analysis and relative densitometric analysis. Each bar in panel B shows the mean ± SD of 3 experiments. ***p<0.001 vs. saline; °°°p<0.001 vs. LPS alone.

The mast cell occurrence in intestinal tissue sections stained with toluidine blue is showed in Fig. 2. The mast cells staining showed that in mice treated with saline and with CBD alone, the mast cell number was comparable; however the treatment of mice with LPS induced a significant increase of mast cell number as compared to saline-treated animals. In the tissue sections of LPS-treated mice and receiving CBD treatment a significant reduction in mast cell number was observed. These results were paralleled to those obtained with western blot analysis for both chymase (Fig. 3A) and MMP-9 (Fig. 3B); in fact the protein expression of those mast cell-derived mediators, were significantly up-regulated in LPS mice and reduced after CBD treatment.

### Effect of CBD in LPS- induced macrophages activation in the mouse intestine

**Figure 4:**
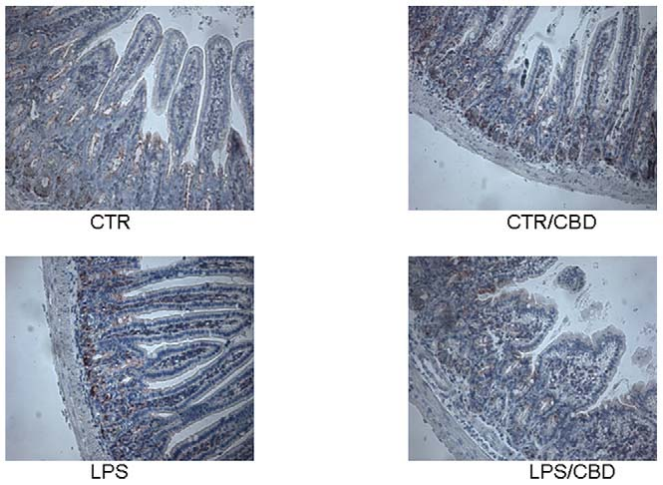
Effect of CBD on macrophages activation in septic mice. Immunohistochemical analysis for MAC3, a know marker of macrophages presence in inflamed tissue, revealed a reduced number of macrophages in intestinal tissues of CBD treated mice.

In intestinal biopsies from control and CBD-treated mice, a mild immunoreactivity for MAC-3, a well established marker of macrophages, was observed, contrarily to the strong and diffuse MAC3 immunoreactivity present in the intestine of LPS-treated mice. However, when mice receiving LPS were treated with CBD MAC3 immunostaining significantly decreased (Fig. 4).

### Effect of CBD in LPS-induced intestinal TNF-α expression in the mouse intestine

**Figure 5:**
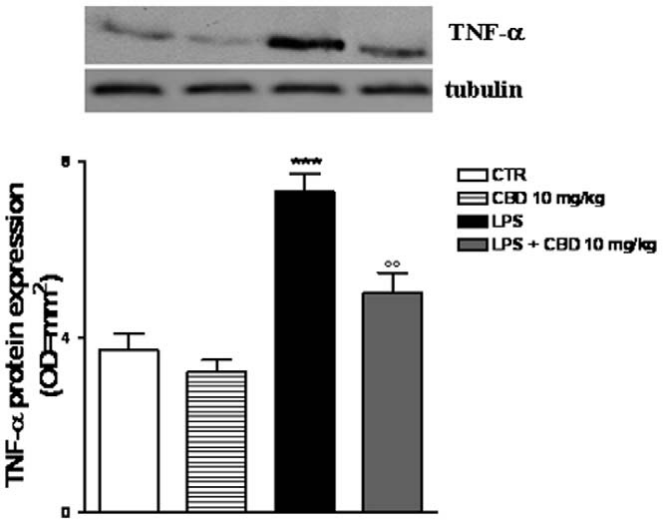
Effect of CBD in LPS-induced intestinal TNF-α expression in the mouse intestine. Western blot analysis showing the effect of CBD on LPS induced TNF-α expression. Panel *(a)* shows TNF-α protein expression; *(b)* densitometric analysis of corresponding bands (optical density). Panel A is representative of *n* = 3 separated experiments. Each bar in panel B shows the mean ± SD of 3 experiments. ***p<0.001 vs. saline; °°°p<0.001 vs. LPS alone.

Immunoblotting analysis of mouse intestinal homogenates demonstrated that TNF-α protein expression was significantly enhanced in mice treated with LPS compared to control mice. CBD treatment *per se* did not affect TNF-α expression, but almost completely reversed the increased levels TNF-α observed in LPS-treated mice (Fig. 5).

### Effect of CBD in LPS- induced apoptosis in the mouse intestine

**Figure 6:**
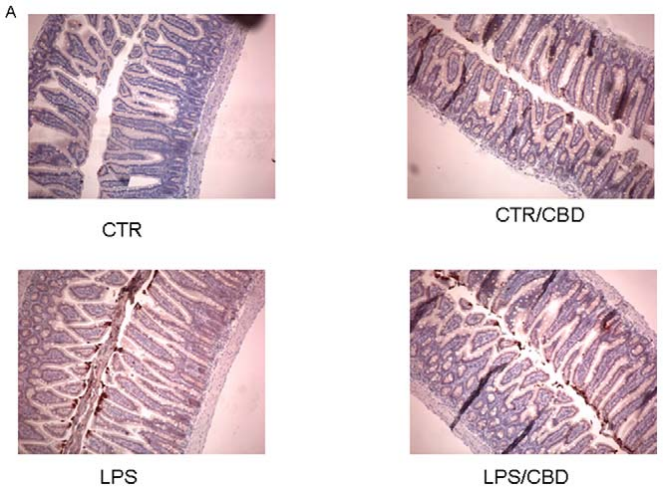
Effect of CBD on caspase-3 activation. (A) Immunohistochemical analysis for cleaved-caspase-3 showed a reduced number of caspase3 positive cells in intestinal tissues of CBD treated mice in confront to LPS mice.

Immunohistochemical analysis revealed that the expression of cleaved-caspase 3, the active form of this pro-apoptotic enzyme, was weakly expressed in intestinal biopsies from control mice and control-mice treated with CBD. In contrast, a strong and diffuse cleaved-caspase 3 immunoreactivity was present in the intestine of LPS-treated mice. Treatment of LPS-mice with CBD significantly reduced the immunoreactivity for cleaved-caspase 3 (Fig. 6).

### Effect of CBD in rectal biopsies of patients with ulcerative colitis (UC) in the remission phase

**Figure 7:**
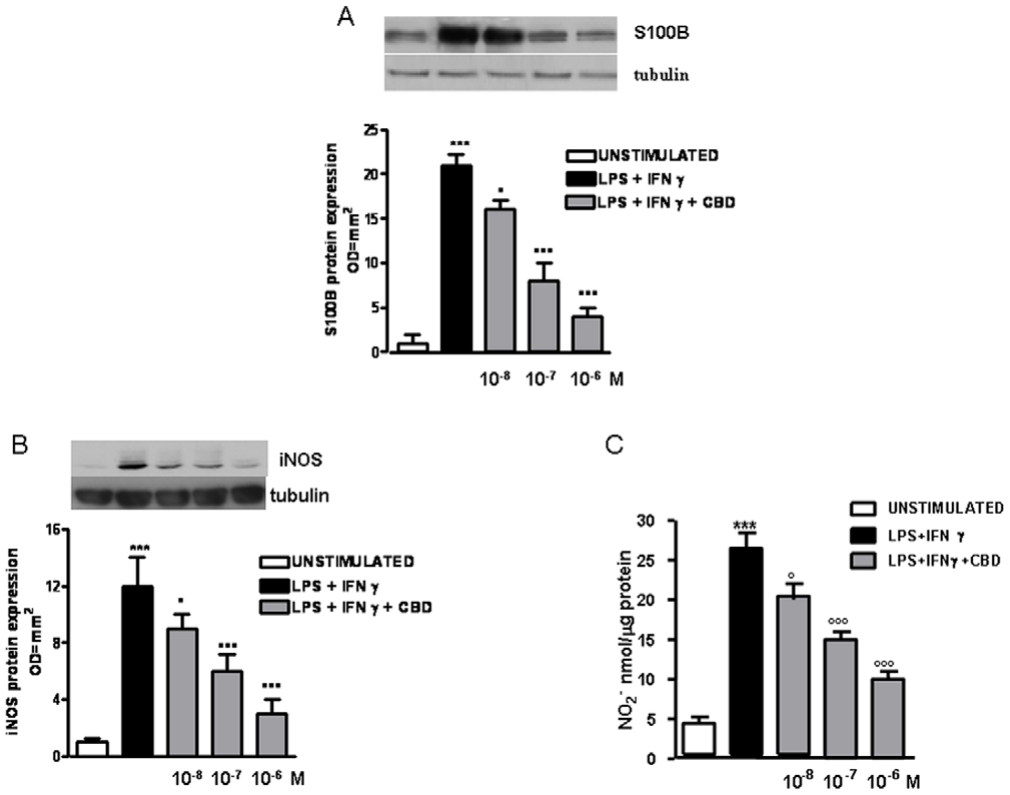
Effect of CBD in rectal biopsies of patients with ulcerative colitis (UC) in the remission phase. (A) shows the effect of CBD on glial activation evaluated as S100B protein expression. Western blot and relative densitometric analysis evidenced that CBD significantly and in a concentration dependent manner reduced LPS- induced S100B. (B) shows the effect of CBD on LPS + INF-γ induced iNOS protein expression and (C) nitrate production. Results are expressed as mean ± SEM of 3 experiments ***p<0.001 vs. CTR; °p<0.05, °°°p<0.001 vs. LPS+INF-γ.

When rectal biopsies from patients with UC in remission phase were cultured for 24 h at 37°C in the presence of LPS plus IFN-γ, S100B and iNOS protein expression were significantly increased in comparison with un-stimulated biopsies from UC patients (Fig. 7A and 7B). Pre-treatment of UC biopsies with CBD significantly reduced, in a dose-dependent manner LPS plus IFN-γ induced iNOS protein expression. Moreover, CBD pre-treatment significantly and concentration-dependently prevented LPS plus INF-γ induced nitrite levels, the stable metabolite of NO (Fig. 7C).

### Effect of CBD in rectal biopsies of patients with ulcerative colitis (UC) in the acute phase

**Figure 8:**
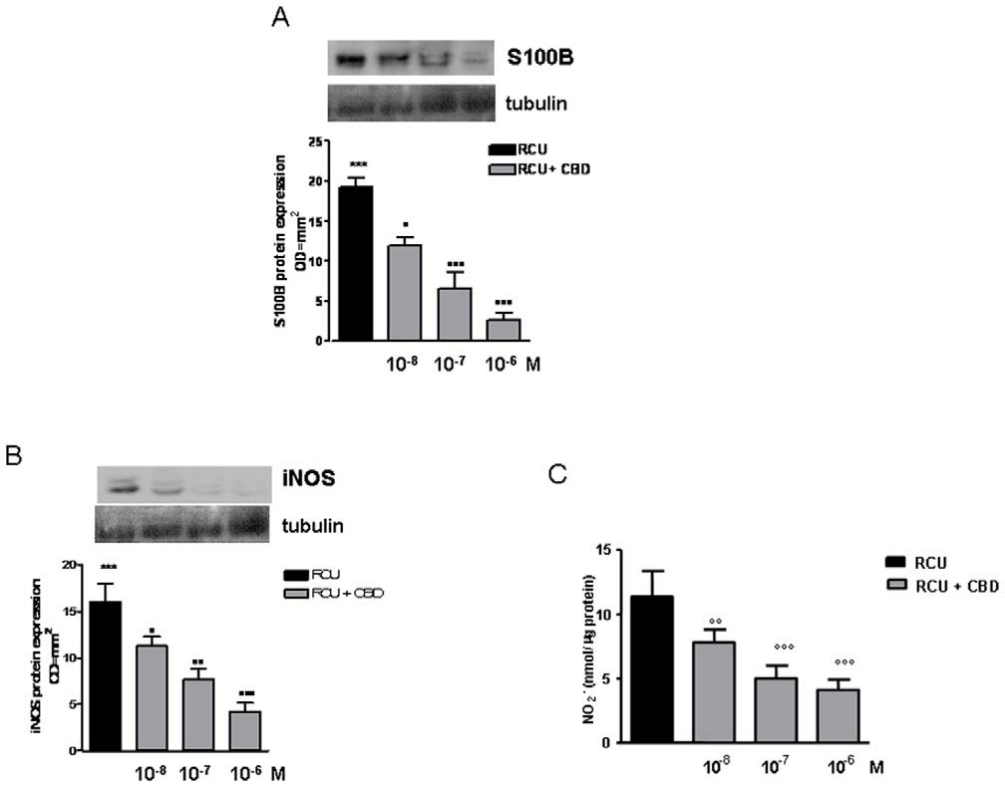
Effect of CBD in rectal biopsies of patients with ulcerative colitis (UC) in the acute phase. (A) shows the effect of CBD on glial activation evaluated as S100B protein expression. Western blot and relative densitometric analysis evidenced that CBD significantly and in a concentration dependent manner S100B expression in biopsies of UC in acute phase. (B) shows the effect of CBD on iNOS protein expression and (C) nitrate production. Results are expressed as mean ± SEM of 3 experiments; °p<0.05, °°°p<0.001 vs. untreated biopsies.

Immunoblotting analysis of un-stimulated rectal biopsies from UC patients in the acute phase revealed that S100B and iNOS protein expression were increased. The expression of both S100B and iNOS proteins was significantly and concentration-dependently reduced in biopsies treated with CBD for 24 h (Fig. 8A and 8B).

In parallel, the administration of CBD to UC biopsies in acute phase significantly inhibited nitrite production (Fig. 8C).

### Effect of CBD and GW9662 in rectal biopsies of patients with ulcerative colitis (UC) in the acute phase

**Figure 9:**
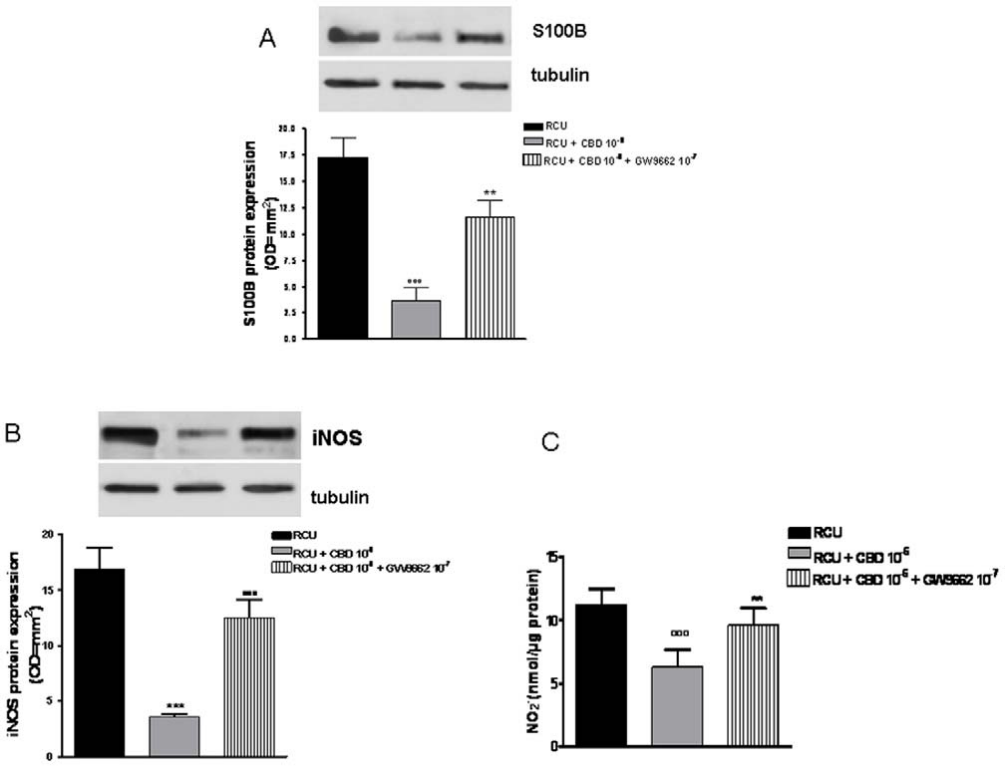
Effect of CBD and GW9662 in rectal biopsies of patients with ulcerative colitis (UC) in the acute phase. (A) shows the effect of CBD and GW9662 on glial activation evaluated as S100B protein expression. Western blot and relative densitometric analysis evidenced that GW9662 significantly counteracts the effect of CBD on S100B expression in biopsies of UC in acute phase. (B) shows the effect of GW9662 and CBD on iNOS protein expression and (C) nitrate production. Results are expressed as mean ± SEM of 3 experiments; °°°p<0.001 vs. untreated biopsies; **p<0.05, ***p<0.001 vs CBD.

The administration of GW9662, a potent PPAR-γ antagonist, significantly reversed the effect of CBD on glial cell activation observed in UC biopsies in acute phase, as showed by the decreased levels of S100B, a marker of glial cell activation. In parallel, GW9662 counteracted also the previously showed effects of CBD on iNOS protein expression and NO production (Fig. 9).

## Discussion

In the present study we investigated the effect of CBD, as a possible modulator of the gut neuro-immune axis, based on its ability to control both inflammatory response, during intestinal inflammation, and EGC activation. EGC maintain the integrity of gut mucosa and act as immuno-competent cells against pathogenic stimuli [17]. They also may trigger and perpetuate gut inflammation by the set up of a complex cross talk with other immune system cell types [18]. More recently, enteroglial activation, through S100B up-regulation, was described in the rectal mucosa of patients with UC [8], suggesting that pharmacological modulation of glial responses may represent a novel therapeutic approach for intestinal inflammatory pathologies.

In line with this, we found that the LPS-induced inflammatory response in mice intestine resulted in an increased expression of S100B, a protein that is exclusively localized in glial cells [19], [20]. Interestingly, pre-treatment with CBD was able to prevent glial cell hyper-activation in the intestine of LPS mice, as revealed by the decreased expression of S100B. In agreement with this theory, we found that the decline of S100B expression after CBD treatment was paralleled by a decreased presence of inflammatory cells in the intestine of LPS treated mice. Particularly, treatment with CBD prevented the LPS-induced increase in the number of mast cell, as underlined by histological analysis and the measurement of chymase expression, an enzyme that is selectively contained in mast cell granules. Our results suggest that, by controlling mast cells, CBD is able to modulate the immune response to inflammation. Following activation, mast cells trigger the recruitment of immune cells, among them neutrophils, to the site of infection [21], [15]. In agreement with this theory, we demonstrated that CBD also reduced the presence of macrophages, as shown by immuno-histochemistry for MAC3, a key marker of macrophages. Moreover, CBD reduced the LPS-induced over-expression of several important mediators such as TNF-α and chymase. Multiple studies have shown that an increase of intestinal damage is associated with an alteration in cytokine levels [22], [23] and an increase of gut epithelial apoptosis [24], [25]. This agrees well with our results showing that the treatment with CBD accounts for a protection of intestinal damage as revealed by analysis of caspase-3 activation. Taken together all these results suggest, for the first time, that CBD, by modulating the glial-immune axis, regulates the fire up of the inflammatory reaction in the intestine thereby preventing the detrimental intestinal damage.

The deregulation of the ENS not only takes place in the acute phase of inflammation but is also considered as a promoter event of chronic inflammation [26], [27]. Therefore, in the second part of this work we focussed on the effect of CBD in human intestinal biopsies from patients with ulcerative colitis, a chronic intestinal inflammatory condition.

Our data evidenced that the administration of CBD in cultured biopsies of UC patients in remission phase was able to reduce the expression of S100B. A recent study by Cirillo et al., [8] indicates that cultured biopsies stimulated with LPS+INF-γ represent an adequate *ex vivo* model for the study of EGCs involvement during UC. According to this theory, the pre-treatment of LPS/INF-γ biopsies with CBD significantly prevents the expression of S100B, and therefore the activation of EGC. The reduced enterogliosis was accompanied with a lower environment in cultured biopsies, as revealed by the reduction of iNOS and NO production, two key markers of the inflammatory status [20].

The effects of CBD were confirmed in a second set of experiments, in biopsies from UC patients in active phase. The administration of CBD in cultured biopsies significantly reduced the expression of S100B and, in parallel, decreased the severity of intestinal inflammation.

Finally, we intended to better elucidate the mechanism of CBD action. Although CBD does not directly interact with CB1 or CB2 receptors, in our previously study we demonstrated that CBD indirectly interacts with cannabinoid receptors through the modulation of the endocannabinoid system in septic mice [14]. In accordance with the recent literature proposing a direct interaction of CBD with PPAR-γ receptor [12], [13], in the present paper, therefore, we tried to counteract the effect of CBD with a PPAR-γ antagonist. Our results indicate that the administration of GW9662, a potent PPAR-γ antagonist, partially reversed CBD effects first of all on S100B production and consequently also on parameters (iNOS and NO) in acute phase of UC. This result is perfectly in line with the important role played by PPAR-γ receptors during intestinal inflammation [28].

Our results indicate that CBD is a key modulator molecule that may interfere with the enteroglial-mediated interactions in an intestinal inflammatory environment. Its activity, markedly focused on S100B protein downregulation, leads to consequent reduction of intestinal damage occurring during acute and chronic intestinal inflammatory status and highlights the importance of glial cells control during these pathological conditions.

The results of the present study correlate and expand the findings suggesting CBD as a potent compound that is able to modulate experimental gut inflammation [29], [30]. The exact cellular signalling pathways responsible for the effect of CBD still remain unclear, even though, for the first time, we identified PPAR-γ as a key receptor in its action during gut inflammation.

However, in this study we demonstrate that during intestinal inflammation, CBD is able to control the inflammatory scenario and the subsequent intestinal apoptosis through the restoration of the altered glia-immune homeostasis. CBD is therefore regarded as a promising therapeutic agent that modulates the neuro-immune axis, which can be recognised as a new target in the treatment of inflammatory bowel disorders.
